# Mutations in the P10 region of procaspase-8 lead to chemotherapy resistance in acute myeloid leukemia by impairing procaspase-8 dimerization

**DOI:** 10.1038/s41419-018-0511-3

**Published:** 2018-05-03

**Authors:** Ming Li, Xiao-Mo Wu, Ju Gao, Fen Yang, Cui-Lin Zhang, Kun Ke, Ying-Chao Wang, You-Shi Zheng, Jian-Feng Yao, Ying-Ying Guan, Xuan Chen, Juan Chen, Xiao-Long Liu, Xiao-Yu Yang

**Affiliations:** 1 The School of Basic Medical Sciences, Fujian Medical University, Fuzhou, 350108 People’s Republic of China; 2 Department of Biomedicine, University of Basel, Basel, 4056 Switzerland; 3 The United Innovation of Mengchao Hepatobiliary Technology Key Laboratory of Fujian Province, Mengchao Hepatobiliary Hospital of Fujian Medical University, Fuzhou, 350002 People’s Republic of China; 4 The First People’s Hospital of Huaihua, Huaihua, 418000 People’s Republic of China; 5 Fuzhou Maternity and Child Healthcare Hospital, Fuzhou, 350005 People’s Republic of China

## Abstract

Caspase-8 activation initiates apoptotic signaling cascades, and certain mutations in procasepase-8 have been reported to be associated with the progression and prognosis of different types of tumors. In this study, we have identified four novel mutations, which are highly correlated with chemotherapy resistance and poor prognosis of acute myeloid leukemia (AML) patients, within the P10 subunit of procaspase-8. These newly discovered mutations cause premature termination of translation, resulting in truncated procaspase-8 protein, which is incapable of forming dimer to initiate apoptosis signaling pathway. Further biochemical analysis reveals that the segment of P10 subunit of procaspase-8 consisting of three amino acid residues from L491 to F493 is crucial for the formation of procaspase-8 interdimer, and the aberration of this segment disrupts the dimerization and consequently precludes the activation of caspase-8 and downstream apoptotic signaling pathway. Therefore, the patients with AML who bear these types of P10 mutations were more likely to develop chemotherapy resistance due to impaired apoptotic signaling in cellular system, leading to significantly reduced overall survival (OS) as compared with patients carrying no such types of P10 mutations. Taken together, these newly identified P10 mutations in procaspase-8 could be used as novel biomarkers for predicting response and survival of chemotherapy-treated AML patients, as well as potential therapeutic targets for medical intervention in the future.

## Introduction

Acute myeloid leukemia (AML) is characterized by the rapid growth of abnormal white blood cells (WBCs), which interferes with normal blood cell production and differentiation in the bone marrow, and it is the most common type of leukemia diagnosed in adults and children, accounting for ~ 1.3% of all new cancer cases per year^1^. To date, the 5-year survival of AML patients is only around 27%^1^. Chemotherapy remains the first-line treatment for AML patients, and approximately 40–50% of young patients and 10–20% of elderly patients can be cured with conventional chemotherapeutic intervention^2^; however, still 20–30% of young patients and 40–50% of elderly patients do not respond to chemotherapy while experience primary induction failure^3^. In addition, a substantial number of patients who have achieved complete remission (CR) with initial chemotherapy eventually relapse, and the prognostic outcome for patients with recurrence is very poor, with an estimated 5-year survival rate of approximately 11%^4^. Several studies have suggested that AML cancerous cells developed insensitivity and resistance to chemotherapeutic agents^5^, which should induce tumor cell apoptosis during treatment^6^.

The caspase family comprising a group of aspartate-specific cysteine proteases, is playing essential roles in apoptosis signaling. Caspase-8 is an activated form of procaspase-8, and produced by the proteolytic cleavage of procaspase-8 dimer to serve as an initiator of the sequential activation of caspases in the apoptotic signaling cascade^7^. Caspase-8-deficient cells are found to be resistant to apoptosis. The procaspase-8 gene consists of 11 exons, and encodes an inactive pro-enzyme (479 aa) composed of a prodomain and a caspase domain. The prodomain comprises two death-effector domains (DEDs) which are capable of interacting with the Fas-associated death domain to form the death-inducing signal complex^8^. The caspase domain consists of two subunits known as P18 and P10 (ref^9^), which can be further processed to form the active caspase-8 heterotetramer and then be released into the cytosol to trigger the activation of remaining downstream signaling, leading to apoptosis^10^.

Dysfunction of apoptotic signaling due to an abnormality in procaspase-8 has been implicated in a variety of diseases. For example, aberrant expression of procaspase-8 has been linked to abnormal fetal cardiovascular development and hematopoietic malfunction^11^, and several polymorphisms or mutations in the procaspase-8 gene have been associated with head and neck squamous cell carcinoma^12,13^, vaginal squamous cell carcinoma^14^, lung cancer^15^, hepatocellular carcinoma^16^, gastric carcinoma^17^, colon cancer^18^, and breast cancer^19,20^. To date, all of these reported gene polymorphisms or mutations are located in either the promoter region or the DED domain of procaspase-8. However, disease-associated functional mutations within the caspase domain of procaspase-8, especially in cancer, have not been reported previously.

In this study, 15 novel mutations within the P10 subunit of the caspase domain were identified by sequencing the procaspase-8 gene from the samples of AML patients. Four out of these 15 mutations occurred at considerable high frequency and were discovered to be strongly correlated with high WBC counts and poor prognosis. Further biochemical elucidation revealed that these four mutations undermined the caspase-8-mediated apoptotic pathway by disrupting the formation of dimeric procaspase-8 proteins, resulting in the mutation-harboring cells being relatively insensitive to chemotherapeutic agents. This study provides a better understanding of the heterogeneity of AML cancerous cells and therefore the implication of procaspase-8-mediated apoptosis in chemotherapy treatment further provides potential therapeutic targets for medical intervention of AML patients in the future.

## Results

### Mutation identification in the P10 subunit of procaspase-8 from AML patients and the clinical significance of identified mutations

**Fig. 1:**
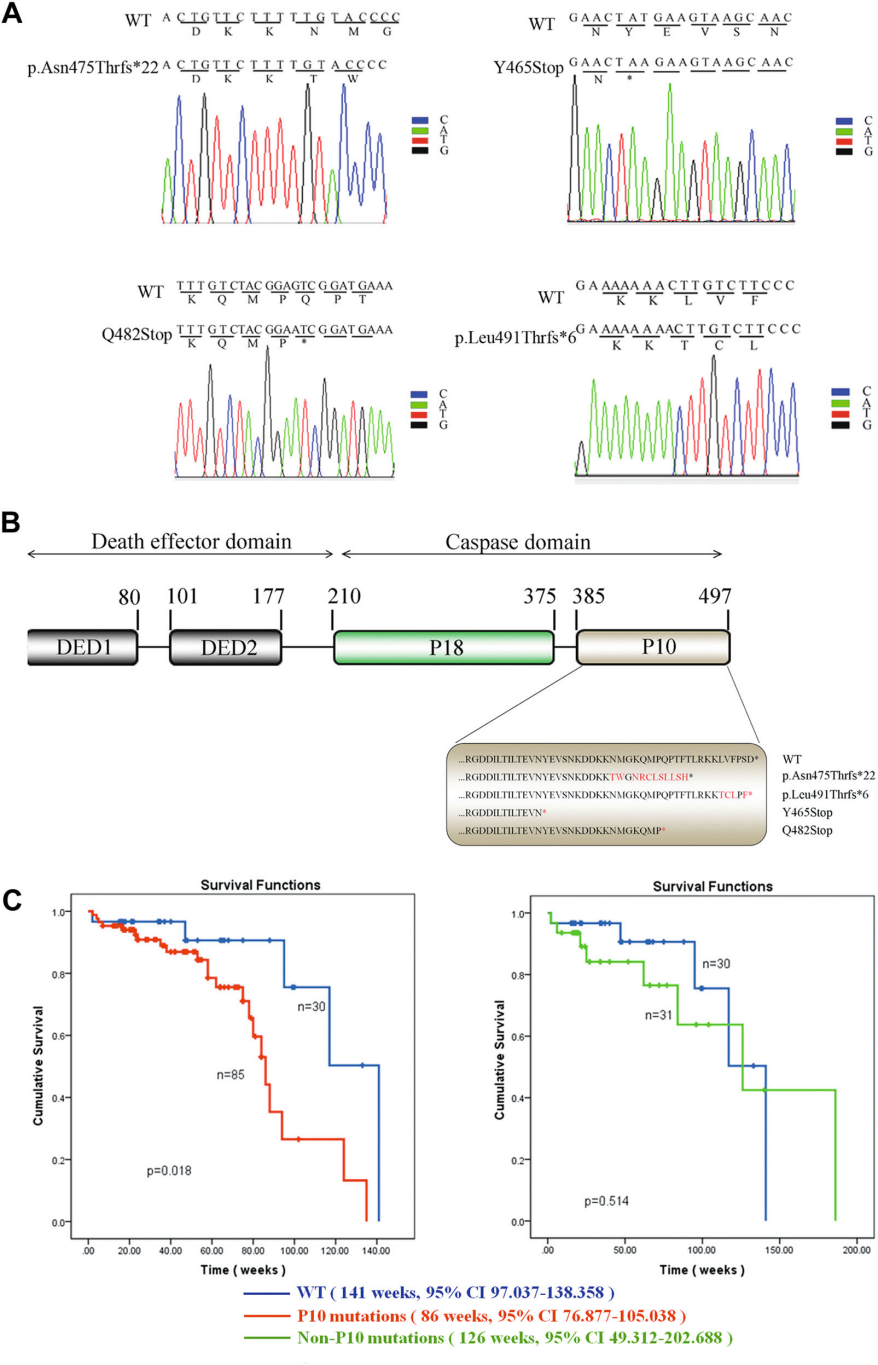
Identified mutations in the P10 subunit of procaspase-8 from AML patients and their clinical significances. **a** DNA sequencing to identify the P10 mutations. Here, 1423_del A, an adenine deletion at 1423 of procaspase-8 that causes a frameshift mutation from Asn to Thr, is denoted as p.Asn475Thrfs*22; the 1395 T > A mutation, a thymine-to-adenine mutation at 1395 of procaspase-8 that produces a stop codon mutation at Tyr, is denoted as Y465Stop; the 1444 C > T mutation, a cytosine-to-thymine mutation at 1444 of procaspase-8 that produces a stop codon mutation at Gln, is denoted as Q482Stop; the 1471 ins A mutation, an adenine insertion at 1471 of procaspase-8 that results in a frameshift mutation from Leu to Thr, is denoted as p.Leu491Thrfs*6. **b** Schematic illustration of the P10 mutations in the procaspase-8 protein. **c** The P10 mutations were closely correlated with short OS for AML patients. OS was significantly different between the patients carrying the P10 mutations and those carrying no mutations in the P10 subunit (left, Kaplan–Meier, *p* = 0.018) and there was no statistical difference between the non-P10 mutation group and the WT group (right, Kaplan–Meier, *p* > 0.05)

To identify potential mutations within the P10 region of procaspase-8, the last exon of procaspase-8 was amplified by PCR and then subjected to Sanger sequencing. In total, 15 mutations were identified in the P10 regions out of 146 AML patients, and finally 4 missense mutations out of 15 at a considerable high frequency were selected for further investigation: the p.Asn475Thrfs*22 mutation (in 39 AML patients, accounting for 26.71% of the total analyzed population), the Y465Stop mutation (in 14 AML patients, accounting for 9.59% of the total analyzed population), the Q482Stop mutation (in 8 AML patients, accounting for 5.48% of the total analyzed population), and the p.Leu491Thrfs*6 mutation (in 24 AML patients, accounting for 16.43% of the total analyzed population) (Fig. 1a,b). These four mutations are referred as “the P10 mutations” in the following analysis described below.

To examine whether the P10 mutations exerted any effects on the chemotherapy treatment and the prognosis of AML patients, an association analysis between the P10 mutations and the clinical–pathological characteristics of patients was performed. As shown in Table 1, the patients who carried these P10 mutations had significantly higher WBC counts (*p* = 0.00432) and lower CR rates after chemotherapy (15.30% vs. 32.79%; *p* = 0.0162) than the patients without P10 mutations (including patients without P10 mutations but with other mutations and patients with wild-type (WT) procaspase-8); however, such clinical characteristics were not significantly different between the WT patients and the non-P10 mutation patients (without the identified P10 mutations but with other mutations in the P10 region), as shown in Supplementary Table 1. In contrast, characteristics including age, sex, French–American–British subtype, and CR duration (data not shown) were not statistically correlated with the P10 mutations. Meanwhile, to further confirm above observations, the association between clinical–pathological features and well-known mutations (FLT3-ITD, CEPBA, NMP) was also investigated; as shown in Supplementary Table 2, the patients who carried FLT3-ITD mutations also had significantly lower CR rates after chemotherapy than the patients without FLT3-ITD mutations; however, the FLT3-ITD mutations were not significantly associated with the P10 mutations in our current study (Supplementary Table 3). Furthermore, based on long-term follow-up data, the Kaplan–Meier analysis was performed to investigate whether these P10 mutations had any effects on the overall survival (OS), and the patients without P10 mutations in Table 1 were further divided into non-P10 mutations group and the WT group (without any mutations). Therefore, the patients were assigned into three experimental groups. As shown in Fig. 1c, the patients with P10 mutations in the cohort had a significantly shorter median survival time (86 weeks, 95% confidence interval (CI) 76.877–105.038) than the WT patients without mutations in the P10 subunit (141 weeks, 95% CI 97.037–138.358), whereas the patients carrying the non-P10 mutations (126 weeks, 95% CI 49.312–202.688) had no statistically difference comparing with the WT patients. Taken together, these data indicate that the P10 mutations are highly associated with poor prognosis of AML patients after chemotherapy.

**Table 1:** The clinic-pathological features of 146 AML patients

Variables	P10 mutations	Without P10 mutations	*p*-Value
*Age*			
<60 Years	72	45	0.1404
≥60 Years	13	16	
*Gender*			
Male	49	37	0.7338
Female	36	24	
*WBC*			
>10 × 10^9^/L	55	29	0.00432^**^
≤10 × 10^9^/L	30	32	
*Response to chemotherapy*			
Remission	13	20	0.0162^*^
Non-remission	72	41	

*χ*^2^ test, * *p* < 0.05; ** *p* < 0.01

### P10 mutations abolish procaspase-8/caspase-8-mediated apoptosis

**Fig. 2:**
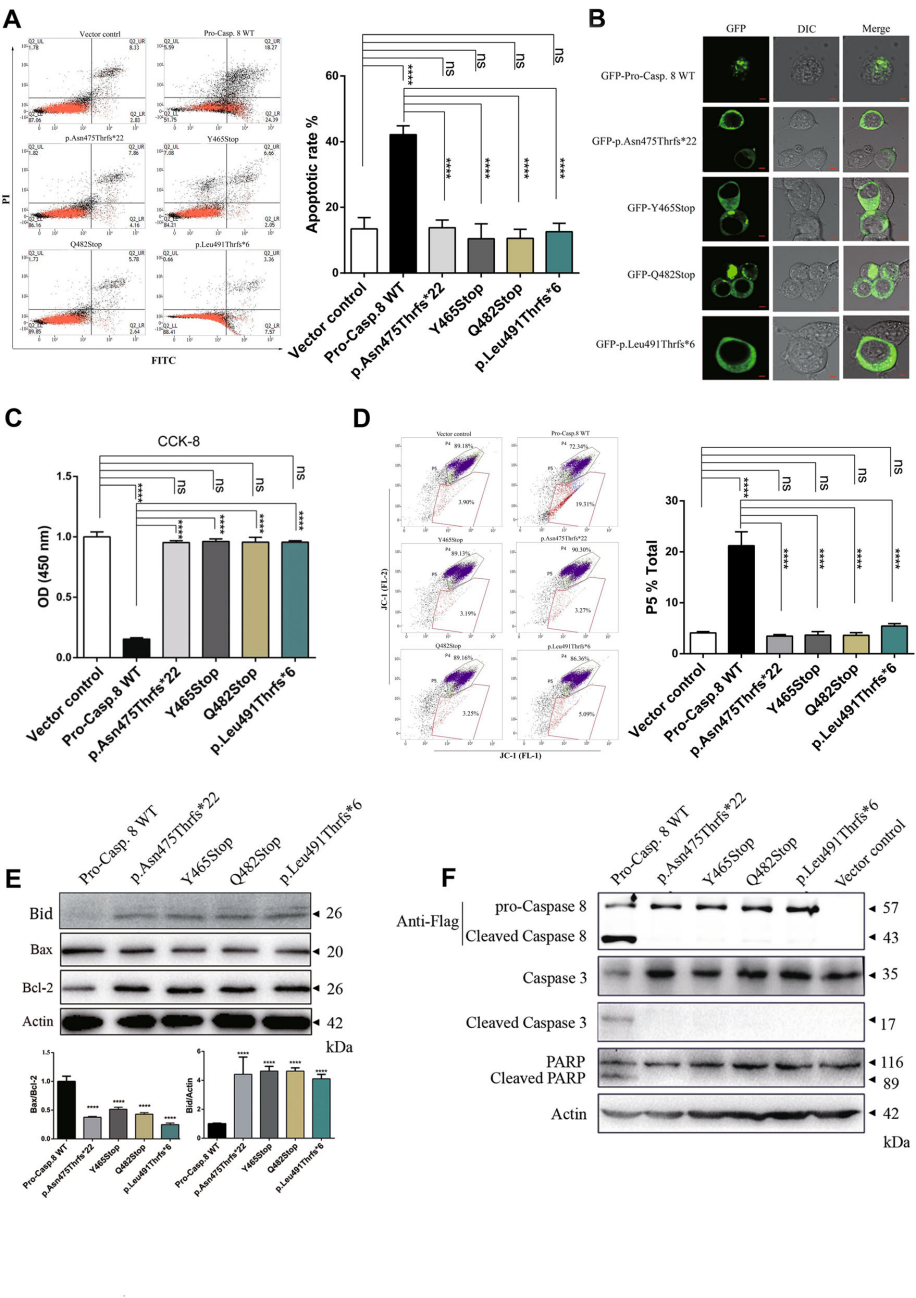
The P10 mutations abolish procaspase-8/caspase-8 mediated apoptosis. **a** Apoptotic rate was measured by flow cytometry via Annexin V–FITC/PI staining. Representative results were displayed on the left. The quantification of three independent experiments was shown on the right. All data were represented as the mean ± SD (one-way ANOVA, ****p < 0.0001, ns, *p* > 0.05). **b** Confocal microscopy was performed to analyze apoptosis in 293T cells. In contrast to the P10 mutations, only the cells expressing GFP-tagged procaspase-8 WT showed apoptotic morphology. Scale bar, 5 μm. **c** Cell viability was assessed with CCK-8 assay. The columns represented the OD values of cells from three independent experiments. All data were represented as the mean ± SD (one-way ANOVA, ****p < 0.0001, ns, *p* > 0.05). **d** Δψ was analyzed by flow cytometry with JC-1 staining. Left: representative results were from three independent experiments. Right: the columns represented the average percentage of cells in P5 from three independent experiments. All data were represented as the mean ± SD (one-way ANOVA, ****p < 0.0001, ns, *p* > 0.05). **e** Immunoblot analysis of Bax, Bcl-2, and Bid. Top: representative results were from three independent experiments. Bottom: the columns represented the mean level of Bax/Bcl2 (left) and Bid/Actin (right) from three independent experiments. All data were represented as the mean ± SD (one-way ANOVA, ****p < 0.0001). **f** Immunoblot analysis of the cleavage of procaspase-8 (bloted by the anti-Flag antibody) and apoptosis-related proteins (caspase-3 and PARP). Representative results were from three independent experiments

To investigate the molecular mechanism underlying the poor clinical outcomes associated with the P10 mutations and to delineate their potential functions in apoptosis, the apoptotic signaling cascade was monitored in the presence or absence of the P10 mutations in 293T cells^21,22^. The green fluorescent protein (GFP)/Flag-tagged procaspase-8 WT and P10 mutant plasmids, as well as the control vector were constructed according to the schematic illustration shown in Supplementary Figure 1A. Furthermore, Annexin V–fluorescein isothiocyanate (FITC)/propidium iodide (PI) staining was performed to detect apoptosis in the presence of the P10 mutations versus procaspase-8 WT in 293T cells. As shown in Figure 2a, the apoptotic rates of cells overexpressing procaspase-8 WT construct were about 40% (42.140 ± 2.718%), which were considerably higher than cells transfected with the control vector (13.463 ± 3.418%, *p* < 0.05); conversely, the apoptotic rates of cells transfected with the P10 mutations were only ~ 10% (13.803 ± 2.345% for p.Asn475Thrfs*22, 10.467 ± 4.519% for Y465Stop, 10.580 ± 2.760% for Q482Stop, and 12.600 ± 2.554% for p.Leu491Thrfs*6), showing no significant differences comparing with the control vector (*p* > 0.3). The cell morphology change was further observed by confocal microscopy to study the apoptosis of each transfected cells. As shown in Figure 2b, the cells overexpressing procaspase-8 WT construct were shrink and showed a typical apoptotic morphology, whereas the cells overexpressing the P10 mutations or the control vector remained intact and showed a normal morphology. Furthermore, the cell viability of each transfected group was determined by the Cell Counting Kit-8 (CCK-8) assay. As shown in Figure 2c, the viability of cells overexpressing procaspase-8 WT was 15.4 ± 1.1% after 24 h of transfection and it is significantly lower than the control vector transfected cells (100 ± 4%, *p* < 0.001), whereas the viabilities of cells transfected with the P10 mutations were 95.3 ± 1.5% (p.Asn475Thrfs*22), 96.2 ± 2.2% (Y465Stop), 95.7 ± 4% (Q482Stop), and 95.6 ± 1.2% (p.Leu491Thrfs*6), respectively, demonstrating no statistical differences compared with the control vector (*p* = 0.1325, 0.2224, 0.2563, and 0.1465, respectively). However, further proliferation analysis showed that these identified P10 mutations had no effects on cell proliferation (Supplementary Figure 1B), indicating that the P10 mutations only abolished the procaspase-8/caspase-8-mediated apoptosis.

Furthermore, mitochondrial outer membrane permeabilization was monitored by measuring the depolarization of the mitochondrial membrane potential (Δψ) via JC-1 staining, to evaluate cellular apoptosis. As shown in Figure 2d, the cells transfected with procaspase-8 WT displayed much higher rate of the Δψ (21.225 ± 2.708%) than the control vector-transfected cells (4.085 ± 0.262%, *p* < 0.01), as well as the P10 mutations-transfected cells (3.475 ± 0.290% for p.Asn475Thrfs*22, 3.665 ± 0.672% for Y465Stop, 3.615 ± 0.516% for Q482Stop, and 5.430 ± 0.481% for p.Leu491Thrfs*6, with *p* < 0.01), whereas the Δψ rate of the control vector-transfected cells and the P10 mutations-transfected cells were quite similar (with *p* > 0.05). Furthermore, in-depth analysis of the related signaling indicators showed the Bax/Bcl-2 ratio was much lower, whereas the remaining Bid level was much higher in the cells with P10 mutations when compared with the procaspase-8WT-expressing cells, indicating the function of attenuating apoptosis by P10 mutations (Fig. 2e).

Next, the apoptotic downstream signaling cascades were further investigated by analyzing the cleavage of procaspase-8, caspase-3, and polyadenosine diphosphate-ribose polymerase (PARP). As shown in Figure 2f, the cells transfected with the P10 mutations failed to produce the cleaved caspase-8 proteins, in contrast with the procaspase-8 WT-transfected cells, where most of the procaspase-8 proteins were proteolyzed to produce the cleaved version of caspase-8. In addition, cleaved caspase-3 and cleaved PARP could only be detected in the presence of procaspase-8 WT but not in the presence of the P10 mutations. Taken together, these results clearly demonstrate that these four mutations within the P10 subunit are capable of abolishing procaspase-8/caspase-8-mediated apoptosis.

### Insensitive to etoposide treatment caused by the P10 mutations

**Fig. 3:**
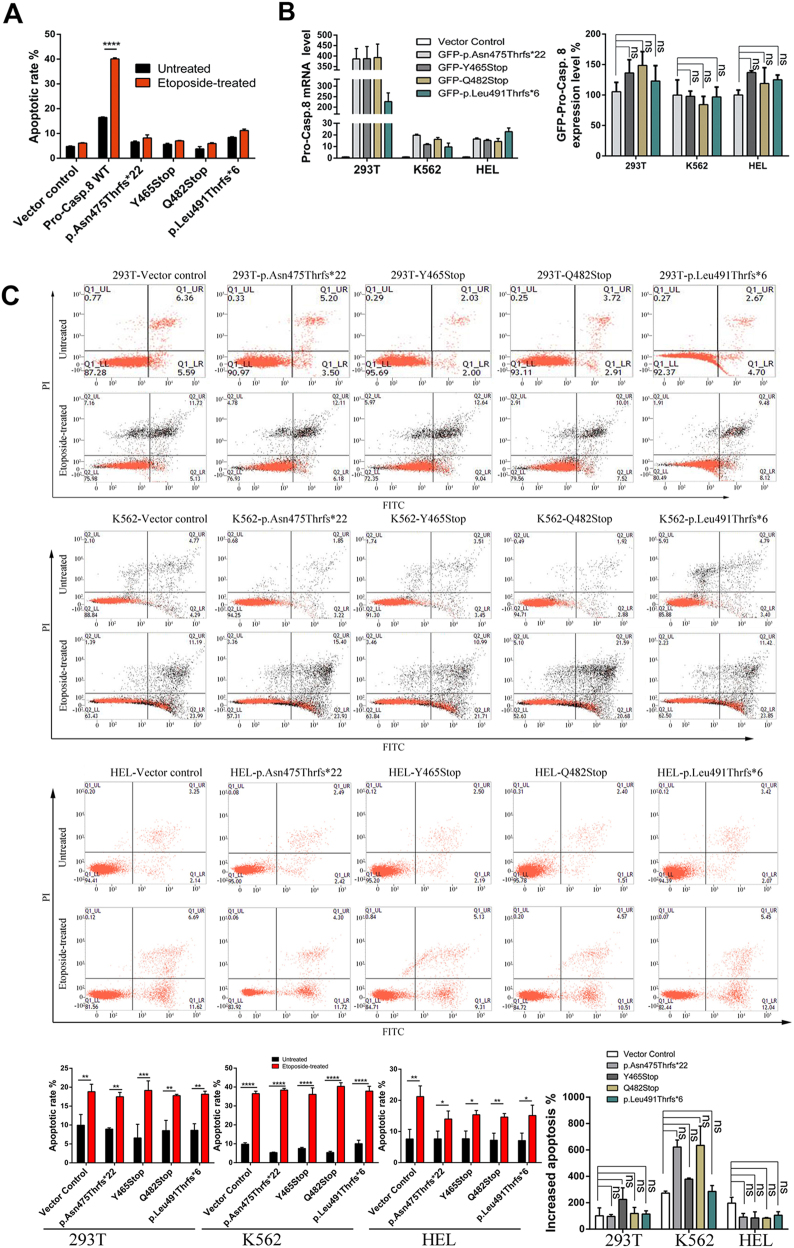
Insensitive to etoposide treatment caused by the P10 mutations. **a** Cell apoptosis was analyzed in 293T cells transfected with the P10 mutations or procaspase-8 WT with or without the etoposide treatment for 8 h. The columns represented the average percentage of Annexin V–FITC-positive cells from three independent experiments. All data were represented as the mean ± SD (two-way ANOVA, Dunnett’s test, ****p < 0.0001). **b** 293T, HEL, and K562 cells were infected with lentivirus containing the protein of interest, and cells were selected with puromycin for 1 week. Left: the mRNA level was quantified by real-time PCR by targeting procaspase-8 as the template. Right: the procaspase-8 expression level was quantified by immunoblotting. The columns represented the average level from three independent experiments. All data were represented as the mean ± SD (one-way ANOVA, ns, *p* > 0.05). **c** Cell apoptosis was analyzed by flow cytometry in stable 293T, HEL, and K562 cell lines treated with or without 100 nM etoposide. Representative results from three independent experiments in the stable 293T (the first panel), K562 (the second panel), and HEL (the third panel) cell lines were displayed. The columns represented the average percentage of Annexin V–FITC-positive cells for the stable 293T, K562, and HEL cell lines. All data were represented as the mean ± SD (*t*-test, two-tailed, **p < 0.01, ***p < 0.001, and ****p < 0.0001). The average increase of apoptotic percentage after etoposide treatment for each group was displayed at the right of the fourth panel and it was derived from three independent experiments. All data were represented as the mean ± SD (two-way ANOVA, Tukey’s test, ns, *p* > 0.05)

The aberrance of apoptotic signaling pathway in cancerous cells has been linked to drug resistance in chemotherapy^23^. To investigate the potential role of the P10 mutations in chemotherapy-associated apoptotic dysfunction, cellular responses to apoptotic stimuli, such as the chemotherapeutic agent etoposide and hydrogen peroxide (H_2_O_2_), were carefully analyzed. As shown in Figure 3a and Supplementary Figure 1C, the etoposide treatment significantly increased the apoptotic rates by nearly 2.28-folds in procaspase-8 WT-transfected cells, whereas the same etoposide treatment nearly failed to induce cell apoptosis in the P10 mutations-transfected cells or in the control vector-transfected cells, probably due to the relatively low concentration of etoposide used in this study. Taken together, these results suggested that the P10 mutations in procaspase-8 rendered the cells insensitive to chemotherapy treatment such as etoposide. It is worth noting that in an attempt to clearly observe the drug-induced apoptosis increasing and to avoid saturation of apoptosis in this study, we hereby reduced the expression level of each construct by using half of the DNA amount in previously experiments; consequently, the apoptotic rates before etoposide treatment were much lower than previously described in the characterization of P10 mutations (Fig. 2a).

Furthermore, to understand whether procaspase-8 proteins with P10 mutations are implicated in response to chemotherapy, myelogenous leukemia-derived cell line known as K-562 and AML cell line of HEL were employed for apoptotic functional assays. We established K562, HEL, and 293T stable cell lines expressing GFP/Flag-tagged P10 mutations and GFP/Flag control construct through lentivirus delivering system. Due to the lethality caused by constitutive expression of procaspase-8, the stable cell line of procaspase-8 WT could not be obtained and was excluded in the following experiments. Both the transcription and expression levels were subsequently examined in each established stable cell line to monitor the expression efficiency of the proteins of interest, and as shown in Figure 3b, the P10 mutants were all equally expressed in K562, HEL, and 293T cells. Subsequently, the K562, HEL, and 293T stable cells were treated with etoposide^24^ (100 nM) for 8 h and then the cell apoptosis was evaluated by flow cytometry. As shown in Figure 3c, the apoptotic rates of the P10 mutants expressing K562, HEL, and 293T cells after etoposide treatment were quite comparable and have no significant differences to those of the control cells (*p* > 0.05).

**Fig. 4:**
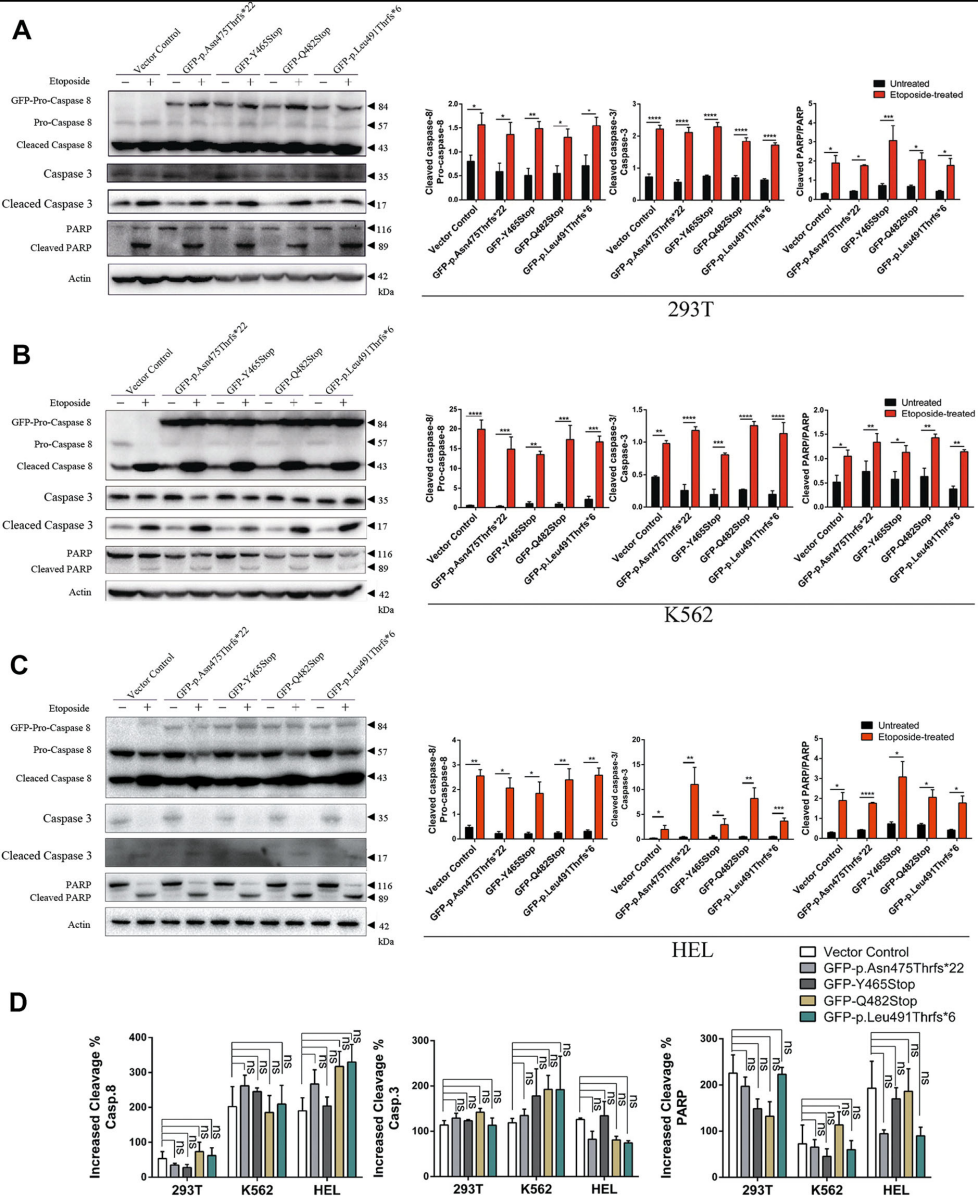
Inhibition of the proteolytic of procaspase-8 protein by the P10 mutations. The **(a)** 293T, **(b)** K562, and **(c)** HEL stable cell lines were treated with or without 100 nM etoposide for 8 h. After treatment, the indicated proteins were detected by immunoblot. Left: representative results were from three independent experiments (− : untreated, + : etoposide-treated). Right: the quantifications of the cleaved caspase-8, caspase-3, and PARP proteins were analyzed from three independent experiments. All data were represented as the mean ± SD (*t*-test, two-tailed, *p < 0.05, **p < 0.01, ***p < 0.001, and ****p < 0.0001). **d** The increased cleavage percentage of caspase-8, caspase-3, and PARP were analyzed in each group after treatment. All data were represented as the mean ± SD (two-way ANOVA, Tukey’s test, ns, *p* > 0.05)

The downstream apoptotic signaling cascade in response to the etoposide treatment was also investigated. As shown in the Figure 4, the etoposide treatment led to increased proteolysis of endogenous procaspase-8 and accordingly increased cleavage of caspase-3 and the PARP protein in all of the K562, HEL, and 293T cell lines. However, the etoposide treatment could not trigger the proteolytic cleavage of exogenous procaspase-8 proteins carrying P10 mutations and, consequently, there was no amplification of the downstream signaling cascade due to the presence of procaspase-8 P10 mutations. Consistent with the response to etoposide treatment, the H_2_O_2_ treatment neither additionally enhanced apoptosis induction nor triggered the cleavage of the procaspase-8 P10 mutants (Supplementary Figures 2 and 3).

In summary, these results demonstrated that the P10 mutations in procaspase-8 rendered the cells being insensitive to etoposide treatment and suggested the possible molecular mechanism underlying the poor clinical outcomes to chemotherapy and drug resistance that were observed in AML patients carrying these P10 mutations.

### P10 mutations in procaspase-8 inhibit dimer formation

**Fig. 5:**
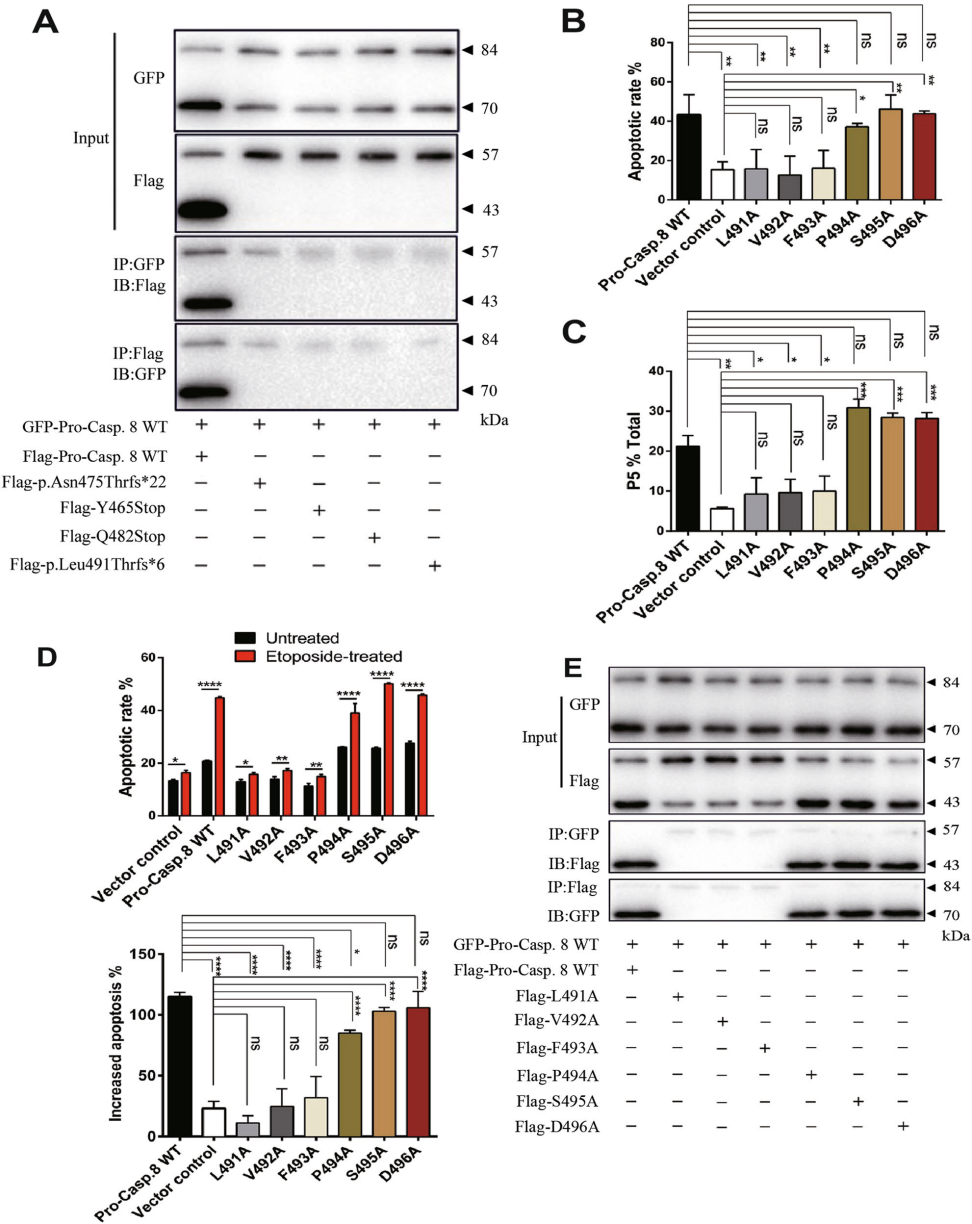
Inhibition of the dimerization of procaspase-8 by the P10 mutations. **a** 293T cells cotransfected with GFP-tagged and Flag-tagged procaspase-8 WT or the P10 mutations. After 24 h of transfection, total cell lysates were examined by IP as described in Materials and Methods. Representative results were from three independent experiments. **b** Cell apoptosis and **c** Δψ were monitored by flow cytometry. The columns represented the average percentage of Annexin V–FITC-positive cells and the average percentage of P5 from three independent experiments. All data were represented as the mean ± SD (one-way ANOVA, *p < 0.05, **p < 0.01, ***p < 0.001, ns, *p* > 0.05). **d** 293T cells expressing the indicated proteins were treated with or without 100 nM etoposide for 8 h. Apoptosis was assessed by flow cytometry. The columns represented the average percentage of Annexin V–FITC-positive cells (top) and increased apoptosis (bottom) from three independent experiments. All data were represented as the mean ± SD (Top: t-test, two-tailed, *p < 0.05, **p < 0.01, ***p < 0.001 and ****p < 0.0001; Bottom: two-way ANOVA, Dunnett’s test, *p < 0.05, **p < 0.01, ****p < 0.001, ns, *p* > 0.05). **e** 293T cells were co-transfected with GFP-tagged and Flag-tagged WT or indicated procaspase-8 substitution plasmids. After 24 h of transfection, total cell lysates were examined by IP as described in Materials and Methods. Representative results were from three independent experiments

Dimerization of two procaspase-8 monomers is crucial for exposing the hydrolysis site via conformational change and producing the activated caspase-8 protein through self-cleavage to initiate apoptosis signaling^25^. The above results demonstrated the P10 mutations inhibiting the activation of procaspase-8, but the underlying mechanism needs to be further explored. To investigate whether the P10 mutations inhibit procaspase-8 activation by preventing the dimerization of procaspase-8, co-immunoprecipitation (Co-IP) assay was performed to examine the interaction between procaspase-8 WT and the P10 mutants. As shown in Figure 5a, in contrast to the strong dimerization of the procaspase-8 WT proteins, the interaction between the procaspase-8 P10 mutants and the procaspase-8 WT proteins can not be detected, indicating that the P10 mutations disrupted the dimerization of the procaspase-8 monomers.

As L491-D496 was a segment deleted in all P10 mutations, to delineate the role of each amino acid in the dimerization of procaspase-8, site-directed mutagenesis was performed to convert each residue within the L491-D496 segment to alanine (Ala, A) and then the constructs were transfected into 293T cells for further analysis. First, the apoptotic rates of these transiently transfected cells were analyzed by Annexin V–FITC/PI staining. As shown in Figure 5b and Supplementary Figure 4A, the apoptotic rates of 293T cells expressing L491A, V492A, and F493A (15.853 ± 5.784%, 12.613 ± 5.684%, and 16.073 ± 7.192%, respectively) were not significantly different from that of vector controlled cells (16.770 ± 6.282%, *p* > 0.5) but much lower than the procaspase-8 WT-expressing cells (43.327 ± 10.244%, *p* < 0.01), suggesting that the L491A, V492A, and F493A mutations could abrogate procaspase-8/caspase-8-mediated apoptosis. However, the apoptotic rates of P494A-, S495A-, and D496A-expressing cells (37.143 ± 1.749%, 46.150 ± 7.272%, and 43.833 ± 1.423%, respectively) were quite similar to that of WT-expressing cells (*p* > 0.5), but significantly higher than that of the control 293T cells (*p* < 0.05), suggesting that the P494A, S495A, and D496A mutations retained the capability of apoptotic induction and these substitutions had no effects on the function of procaspase-8/caspase-8 signaling. Meanwhile, the effects of these L491-D496 substitutions on Δψ were also investigated. As shown in Figure 5c and Supplementary Figure 4B, the rates of Δψ in the L491A, V492A, and F493A mutation-expressing cells (9.255 ± 4.094%, 9.620 ± 3.323%, and 9.990 ± 3.790%, respectively) were not significantly different from that of the control cells (5.585 ± 0.445%, *p* > 0.2) but much lower than that of the procaspase-8 WT-expressing cells (p < 0.01), whereas those of the P494A, S495A, and D496A substitutions (30.875 ± 2.157%, 28.450 ± 1.103%, and 28.170 ± 1.470%, respectively) were comparable to procaspase-8 WT-expressing cells (21.225 ± 2.708%, *p* > 0.05) but significantly higher than the control cells (*p* < 0.01).

Furthermore, we examined whether etoposide-induced apoptosis was affected by these substitutions. As shown in Figure 5d and Supplementary Figure 4C, the etoposide treatment although slightly increased the apoptotic rates of the cells transfected with L491A, V492A, and F493A mutations (11.0146 ± 6.160%, 24.646 ± 14.649%, and 31.901 ± 17.392%, respectively), but showed no statistically differences when compared with the increased apoptotic rates of the cells transfected with control vector (23.002 ± 5.902%, *p* > 0.05), whereas the etoposide treatment significantly increased the apoptosis rates of the cells transiently transfected with the procaspase-8 WT vector or the P494A, S495A, and D496A mutations (114.900 ± 3.595%, 84.910 ± 2.390%, 102.966 ± 3.115%, and 105.863 ± 13.433%, respectively) when compared with the increased apoptosis rates of the control vector-transfected cells (*p* < 0.05). Interestingly, in contrast with S495 and D496, the substitution at P494 residue impaired the function of procaspase-8 to some extent, resulting in a statistical difference between procaspase-8 WT-expressing cells and the P494A mutation-expressing cells (*p* < 0.05). Taken together, three residues of procaspase-8, namely, L491, V492, and F493, were shown to be crucial for the procaspase-8/caspase-8-mediated apoptosis signaling pathway.

To verify whether these three residues affect apoptosis by regulating the self-dimerization of procaspase-8, Co-IP assay was further performed. As shown in Figure 5e, in a similar way to procaspase-8 WT, the P494A, S495A, and D496A substitutions of procaspase-8 were capable of dimerizing with the procaspase-8 WT proteins, whereas no dimerization was evident between procaspase-8 WT and procaspase-8 containing either the L491A, V492, or F493A mutation, indicating that the L491A, V492A, and F493A mutations could disrupt the binding ability to procaspase-8 WT. In summary, the P10 mutations in procaspase-8 abolished the ability of initiating apoptotic signaling by impairing the dimerization of procaspase-8 monomers and the L491-F493 segment on the P10 subunit had an essential role in self-dimerization.

## Discussion

Procaspase-8, as the precursor of the initiator enzyme in the apoptotic signaling cascades, harbors various mutations that have been associated with a low chemotherapeutic efficacy and poor prognosis in multiple types of cancers. Here we discovered four mutations within the P10 subunit of procaspase-8 from patients with AML, which were occurring with particularly high frequency accounted for over half of the population in our screen (85 out of 146 patients, 58.21%). The AML patients carrying these four P10 mutations had significantly higher WBC counts and considerably lower CR rates after chemotherapy than their counterparts carrying either the WT allele or non-P10 mutations. In addition, the patients with the P10 mutations had significantly shorter survival time than those without them, indicating these P10 mutations were highly correlated with poor chemotherapeutic response and prognosis in AML.

As procaspase-8/caspase-8 has an important role in apoptotic regulation and signaling, we hypothesized that the impaired apoptosis caused by these P10 mutations was responsible for the clinical observations such as abnormal high levels of WBCs and, consequently, being resistant to chemotherapy in AML treatment. Indeed, the impairment in apoptosis was observed when the P10 mutations were overexpressed in 239T cells. The apoptotic rates of procaspase-8 proteins with the P10 mutations were much lower than that of procaspase-8 WT and no cleavage of the procaspase-8 mutants could be detected. Furthermore, the treatment of apoptotic agent etoposide could not increase apoptotic rates when the P10 mutations were overexpressed, in contrast with procaspase-8 WT where etoposide treatment led to ~ 2.28 folds of increase in apoptosis. Taken together, these P10 mutations identified from the AML patients are capable of abolishing procaspase-8/caspase-8-mediated apoptosis and rendering the cells being insensitive to apoptotic stimulation.

Ideally, it would be better to elucidate the effects of the P10 mutations in etoposide- or H_2_O_2_-induced apoptosis in leukemia-derived stable cells and make comparison between P10 mutants and WT counterpart accordingly. However, due to the lethality of constitutive expression of procaspase-8 in cells, the best scenario thereby was to compare P10 mutants with vector-controlled mock condition. In agreement with our hypothesis, our data suggested the presence of procaspase-8 containing the P10 mutations could not exert any additional effects in apoptotic induction, in response to apoptotic stimuli when compared with that of delivery the control vector.

To explore the mechanism underlying the P10 mutations associated with apoptotic inhibition, the dimerization of procaspase-8 was therefore examined. Compared with the strong dimerization between the procaspase-8 WT proteins, the P10 mutations appeared to disrupt the dimerization between the mutant and WT monomers of procaspase-8. Residue substitution analysis could reveal that each individual residue within the segment of L491-F493 is crucial for the formation of procaspase-8 dimers, whereas the residues within the P494-D496 segment are less important for dimerization. In line with the observations in the dimerization assay, the apoptotic rates of the P494A, S495A, and D496A substitutions were comparable to that of WT procaspase-8, whereas the L491A, V492, and F493A substitutions abolished the procaspase-8/caspase-8-mediated apoptosis.

To advance our understanding of AML relapse and chemotherapeutic resistance, significant efforts have been made so far and numerous studies have shown that aberrations in several genes, such as *MCL-1*^26^, *TET*^27^, *FLT3-ITD*^28–30^, *NPM1*^31–33^, and* CEBPA*^34–37^, are associated with drug resistance in AML treatment. Here we reported novel mutations within the P10 subunit of procaspase-8, which could inhibit the caspase-8-mediated apoptotic pathway by disrupting the dimerization of procaspase-8 proteins. It seems that these P10 mutations resulted in chemotherapy resistance of mutation-harboring cancerous cells and therefore poor prognosis in AML patients. At last, these mutations may serve as new biomarkers for predicting the chemotherapy response and prognosis of AML patients during clinical courses, or even could provide potential therapeutic targets for effective medical intervention in AML management.

## Materials and methods

### Clinical materials and sample preparation

Peripheral blood samples and primary bone marrow specimens were collected from 146 newly diagnosed AML patients who had only received chemotherapy at Hospital of Fujian Medical University between September 2015 and December 2016. The samples were collected during routine diagnostic assessments in accordance with the National Comprehensive Cancer Network Guidelines Version 1.2015 of Panel Members of Acute Myeloid Leukemia Society with details provided in Table 1. The project was approved for all human sample collections and usage by the Institution Review Board of Hospital of Fujian Medical University. Written informed consent was received from each participant at the time of blood collection in this study.

### DNA sequencing

Genomic DNA was isolated from peripheral blood mononuclear cells using the TIANamp Genomic DNA Kit (TIANGEN, Beijing, China). Procaspase-8 was amplified by PCR using PrimeStar Max (TaKaRa, Dalian, China) (forward primer 5′-GATCGGATTCCGCCACCATGGACTTCAGCAGAAAT-3′ and reverse primer 5′-GATCAAGCTTTTAATCAGAAGGGAAGAC-3′), and the final PCR products were used for Sanger sequencing (Sangon Biotech, Shanghai, China).

### Cells culture

Human embryonic kidney cell line (HEK293T, 293T, ATCC) was cultured in Dulbecco’s modified Eagles medium (DMEM; Gibco, CA, USA). Human erytroleukemia cell line (K562, ATCC) and AML cell line (HEL, ATCC) were cultured in MEM (Gibco) and RPMI-1640 (Gibco), respectively. All the medium were supplemented with 10% fetal bovine serum (FBS, Gibco) and 100 IU/ml penicillin–streptomycin (Sigma, Massachusetts, USA), and all cells were cultured at 37 °C in 5% CO_2_ incubator.

### Plasmid generation and transfection

The Flag/GFP-tagged procaspase-8 expression constructs were generated using human procaspase-8 cDNA ligated into the BamH1 and Xba1 sites of pcDNA3.1-N4Flag/NEGFP vector (recombined based on pcDNA3.1), which expressed N-terminally tagged procaspase-8. PCR reactions were performed for all procaspase-8 cDNAs (forward primer: 5′-GATCGGATTCCGCCACCATGGACTTCAGCAGAAAT-3′ and reverse primer 5′-GATCAAGCTTTTAATCAGAAGGGAAGAC-3′ were used, for WT and all P10 mutants).

Site-directed mutations were performed with PCR-based mutagenesis. The Flag-procaspase-8 plasmid, which encoded procaspase-8 WT, was used as a template for site-directed mutagenesis. The mutation site was covered with the following internal primers (5′-GATCGGATTCCGCCACCATGGACTTCAGCAGAAAT-3′ (forward, for all site-directed mutations) and 5′-GATCTCTAGATTAATCAGAAGGGAAGACAGCTTTTTTTCTTAG-3′ (reverse, L491A), 5′-GATCTCTAGATTAATCAGAAGGGAAAGCAGGTTTTTTTCTTAG-3′ (reverse, V492A), 5′-GATCTCTAGATTAATCAGAAGGAGCGACAGGTTTTTTTCTTAG-3′ (reverse, F493A), 5′-GATCTCTAGATTAATCAGAAGCGAAGACAAGTTTTTTTCTTAG-3′ (reverse, P494A), 5′-GATCTCTAGATTAATCAGCAGGGAAGAC-3′ (reverse, S495A), and 5′-GATCTCTAGATTAAGCAGAAGGGAAGAC-3′ (reverse, D496A)). cDNAs containing the site-specific mutations were ligated to the pcDNA3.1-N4Flag vector.

Plasmid DNA (pDNA) was amplified in *Escherichia coli* and purified according to the protocol provided in the E.Z.N.A.^TM^ Plasmid Mini Kit (OMEGA, GA, USA). All constructed plasmids were validated with restriction enzymes and sequence analysis. The pDNAs (2500 ng or 1500 ng/etoposide) were transfected into 293T cells in six-well plates according to the protocol provided along with the Lipofectamine® 3000 transfection reagent (Thermo, MA, USA).

### Generation of recombinant lentivirus particles and procaspase-8 mutant cell lines

All retroviruses were produced by cotransfection of the pLenti 6-Flag/GFP or pLenti 6-Flag/GFP-procaspase-8 mutant expression constructs with packaging plasmids PLP-1, PLP-2, and VSVG as follows: pLenti 6-Flag/GFP or pLenti 6- Flag/GFP-procaspase-8 mutants, PLP-1, PLP-2, and VSVG were cotransfected at a ratio of 3:1:1:1 for a total of 20 μg of DNA into the 293T cell line when cells were 70–80% confluent (optional 70%). The medium was collected after 24, 48, and 96 h of transfection, then concentrated at 1500 g for 30 min at 4 °C, and filtered through a 0.45 μm filter. Afterwards, the supernatant was ultracentrifuged at 30,000 r.p.m. at 4 °C for 2 h (Beckman Optima XPN -100, CA, USA). The recombinant lentivirus was resuspended with appropriate volume of phosphate-buffered saline (PBS). The virus titer was quantified with the HIV-1 p24 ELISA kit (Cell Biolabs, CA, USA). The recombinant lentivirus was stored at -80 °C for further usage.

The 293T, K562, or HEL cells were seeding at 1 × 10^6^ cells (for 293T cells) or 2 × 10^6^ cells (for K562 or HEL cells) per well in six-well plates in the presence of polybrene (5 μg/ml). The amount of recombinant lentivirus was calculated ([multiplicity of infection = 30] × cells/titer), and then certain amount of virus was added to the cells. After 6 h of infection, the medium was changed to fresh medium and the cells were cultured normally. A confluence of 70–80% of infected cells was chosen for further puromycin selection (2 μg/ml, Sigma, MO, USA) for 1 week. The puromycin-resistant pools were cultured in DMEM or MEM containing 10% FBS and antibiotics, and the expression of procaspase-8 was examined by reverse transcriptase–quantitative PCR (T-qPCR). The stable cell line with only GFP/Flag was denoted as the mock control.

### RT-qPCR analysis

To determine whether caspase-8 was overexpressed in the stable pools, the mRNA expression levels were examined by RT-qPCR. Briefly, total RNA was isolated from stable pools using the TransZol Up Plus RNA Kit (TRANSGEN, Beijing, China). The RNA concentration was determined using the NanoDrop ND-2000 spectrophotometer (Thermo). Then, RNA (100 ng) was reverse-transcribed using the Transcriptor First Strand cDNA Synthesis Kit (Roche, Basel, Switzerland) in accordance with the manufacturer’s protocol. Finally, the expression levels of procaspase-8 were analyzed using RT-qPCR with the Bsetar® SybrGreen qPCR Mastermix (DBI, Ludwigshafen, GER) in accordance with the manufacturer’s protocol, using the gene-specific primer sets for human procaspase-8 (forward: 5′-GCTGACTTTCTGCTGGGGAT-3′ and reverse: 5′-GACATCGCTCTCTCAGGCTC-3′) and 18S (forward: 5′-CAGCCACCCGAGATTGAGCA-3′ and reverse: 5′-AGTAGCGACGGGCGGTGTG-3′). The contents of the reactions (20 μl) were as follows: forward primer (10 nM), 0.2 μl; reverse primer (10 nM), 0.2 μl; cDNA, 50 ng; qPCR Mastermix (2 × ), 10 μl; and water, to a final reaction volume of 20 μl. qPCR was performed on the StepOnePlus^TM^ real-time PCR system (AB, MA, USA) with the following parameters: pre-denaturation at 95 °C for 2 min; 40 cycles at 95 °C for 15 s, 60 °C for 20 s, and 72 °C for 20 s (the signature was collected at this step), and a melt curve stage at 95 °C for 15 s, 60 °C for 1 min and 90 °C for 30 s. 18S was selected as an internal control. Raw data handling and quantification were performed with StepOne™ software. The 2^−ΔΔCt^ method^38^ was used to calculated the relative level of target gene (procaspase-8) expression. The following equation was used: fold change = relative quantification of the procaspase-8 P10 mutation groups/relative quantification of the mock group.

### Antibodies

All antibodies were purchased from Cell Signaling Technologies (MA, USA) unless indicated. Antibodies used were as following: anti-caspase8 (1:2000, #9746), anti-caspase3 (1:1000, #9665), anti-cleaved caspase3 (1:1000, #9664 P), anti-cleaved PARP (1:1000, #5625 P), anti-PARP (1:1000, #9532 P), anti-GFP (1:2000, #2956), anti-DYKDDDK (1:1000, Flag, TRANSGEN, HT201-02), anti-Bax (1:1000, #5023), anti-Bcl2 (1:1000, Abcam, ab32124, Cambridge, UK), anti-β-Actin (1:1000, TRANSGEN, HC201-02), anti-mouse (1:5000, #7076P2), and anti-rabbit (1:5000, 7074P2) for immunoblotting and Co-IPs.

### Immunoblotting and CO-IP

For immunoblotting, collected cells transfected with the procaspase-8 WT or P10 mutants, or stable pools were washed in cold PBS at 4 °C and lysed in RIPA buffer (50 mM Tris-HCl pH 7.4, 150 mM NaCl, 1% Triton X-100, and 0.1% SDS) supplemented with 0.1 mM phenylmethylsulfonyl fluoride and a protease inhibitor cocktail (Roche) on ice for 20–30 min. Lysates were centrifuged at 12,000 × *g* for 30 min at 4 °C. Supernatants were collected and the protein concentrations were quantified according to the protocol provided in the Easy II Protein Quantitative Kit (BCA, TRANSGEN) by the SpectraMax® M5 Multi-Mode microplate reader (Molecular Devices, CA, USA). Supernatants were mixed with the same volumes of 2 × loading buffer and boiled for 10 min. Approximately 50 µg of protein was separated by 10% SDS-polyacrylamide gel electrophoresis (PAGE), transferred onto a nitrocellulose membrane, blocked with 5% skim milk (BD, MD, USA) in TBST (20 mM Tris-HCl, 500 mM NaCl pH 7.5, 0.1% Tween-20) at room temperature for 2 h and then incubated with relevant primary antibodies at 4 °C overnight. Subsequently, the membranes were incubated with the corresponding secondary antibodies at room temperature for 2 h. Finally, the results were visualized by chemiluminescence.

For Co-IPs, 293T cells were co-transfected with the GFP-procaspase-8 WT and Flag/Flag-procaspase-8 P10 mutant constructs in 60 mm dishes. Cells were cultured for 24 h and lysed in an IP buffer (50 mM Tris-HCl pH 8.0, 1% NP-40, 150 mM NaCl, 1 mM EDTA) containing a protease inhibitor cocktail on ice for 30 min. Following centrifugation at 13,300 × *g* for 30 min at 4 °C, supernatants were collected. As the input samples, several of the cell lysates were mixed with the same volume of 2 × loading buffer and boiled for 10 min. The remaining cell lysates were precleared by incubation with 1 μg of anti-GFP or anti-Flag antibody at 4 °C overnight. Meanwhile, Dynabeads protein G (Thermo) were washed three times with IP buffer using a magnetic particle concentrator (Thermo). Then, the rinsed beads (40 μl) were added to each sample and incubated for 2–4 h at 4 °C. Finally, the bead–antibody complexes were washed in IP buffer for 3 times, resuspended in 50 μl of 1 × loading buffer, boiled for 10 min, and separated by 10% SDS-PAGE for immunoblotting.

### Apoptosis and mitochondrial membrane potential analysis

The transiently transfected cells were cultured for 24 h after the transfection for further analysis, whereas the stable transfected cells were cultured to 70–80% confluence and then used for further analysis. For the apoptosis induction analysis, the cells were treated with etoposide (Sigma-Aldrich, MO, USA) or H_2_O_2_ (Sigma-Aldrich) for 8 h. Then, all cells were collected for apoptosis analysis by flow cytometry (BD FACSCalibur™, CA, USA) using the Annexin V–FITC/PI Apoptosis Detection Kit (Dojindo, Kumamoto, Japan) according to the manufacturer’s protocol. For the membrane potential analysis, the transfected cells (cells were transfected with the Flag-procaspase-8 WT, Flag-procaspase-8 P10 mutants, or Flag-procaspase-8 L491A/V492A/F493A/P494A/S495A/D496A single site mutation plasmids) were cultured for 24 h; then, the rate of Δψ was measured by flow cytometry using the Mitochondrial Membrane Potential Detection JC-1 Kit (BD, CA, USA) according to the manufacturer’s protocol.

### CCK-8 assay

Cell viability was measured with the CCK-8 (Dojindo) according to the manufacturer’s protocol. Briefly, 293T cells were seeded 5000 cells/well into 96-well plates. At 60–70% confluence, the cells were transfected with the Flag-procaspase-8 WT or Flag-procaspase-8 P10 mutations, or Flag-procaspase-8 L491A/V492A/F493A/P494A/S495A/D496A single site mutation plasmids (100 ng/well) according to the protocol provided along with the Lipofectamine® 3000 transfection reagent. After culture for 24 h, 10 μl of CCK-8 solution was added to each well, and the plate was incubated in incubator (37 °C, 5% CO_2_) for another 1 h. Then, the optical density value was measured at the wavelength of 450 nm on the SpectraMax® M5 Multi-Mode microplate reader to analyze cell viability.

### Statistical analysis

Data were presented as the mean ± SD and analyzed with SPSS 19.0 and GraphPad Prism 6.0. Three independent experiments were performed for all measurements. The differences between two groups were analyzed with Student’s *t*-test. Statistical comparisons of the CR duration and OS were performed using the Kaplan–Meier analysis. *p* < 0.05 was considered as statistical significance.

## Electronic supplementary material

Supplementary Figure legends and Tables

supplementary Figure 1

supplementary Figure 2

supplementary Figure 3

supplementary Figure 4
