# Supplementary material for: Mutations in the P10 region of procaspase-8 lead to chemotherapy resistance in acute myeloid leukemia by impairing procaspase-8 dimerization
Source: Cell Death Dis. 2018 May 3;9(5):516. doi: 10.1038/s41419-018-0511-3 (PMC5938697; doi:10.1038/s41419-018-0511-3)
Supplement: Supplementary file 1 — Supplementary Figure legends and Tables [file 41419_2018_511_MOESM1_ESM.docx]

**Supplementary Figure legends**

**Supplementary Figure 1:** **P10 mutations abolished the initiation of apoptosis signaling**

(**A**) Schematic representation of AML-associated missense or frameshift mutations in procaspase-8 P10 subunits and GFP/Flag-tagged mutational constructs. (**B**) The proliferation analysis of P10 mutations transfected cells by CCK-8 assay. (**C**) 293T cells treatment with 100 nM etoposide or without for 8 hours, cell apoptosis analysis by flow cytometry. Representative results were from 3 independent experiments.

**Supplementary Figure 2:** **The presence of the P10 mutations exerting no additional effect on the induction of apoptosis in response to H_2_O_2_**

(**A**) Pro-Casp. 8 mRNA level was quantified by Real-Time PCR after lentivirus with the Flag-tagged indicated genes and screened by puromycin for 1week. (**B**) The K562, HEL and 293T were treated with different concentration of H_2_O_2_ for 8 hours, cell viability analysis by CCK-8. Columns represented the means OD of stable cell lines from 3 independent experiments; all date were represented as mean ± SD. (**C**) After 880 μM H_2_O_2_ for 8 hours, Cells apoptosis by flow cytometric detection of Annexin V-FITC staining in stable K562, HEL and 293T. Representative results from 3 independent experiments in stable 293T (the first panel ), K562 (the second panel) and HEL (the third panel) were shown. Columns represented the average percentage of Annexin V-FITC positive cells in stable 293T, K562 and HEL cell lines. All data were represented as mean ± SD (t test, two-tailed, ****p<0.0001). Average increased apoptosis percentage after H_2_O_2_ treatment in each group was displayed to the right of fourth panel from 3 independent experiments; all data were represented as mean ± SD (Two-way ANOVA, Tukey’s test, ns. p>0.05).

**Supplementary Figure 3:** **Incapability of proteolysis of procaspase-8 proteins with the P10 mutations**

The stable lines of 293T(**A**), K562 (**B**) and HEL **(C)** cells were treated with or without 880 μM H_2_O_2_ for 8 hours. After treatment, the indicated proteins were detected by WB. Left: representative results were from 3 independent experiments (-: untreated, +: H_2_O_2_-treated). Right: the quantifications of the cleaved caspase-8, caspase-3 and PARP proteins were analyzed from 3 independent experiments. All data were represented as mean ± SD (t test, two-tailed, *p<0.05, **p<0.01, ***p<0.001 & ****p<0.0001). (**D**) The increased cleavage percentage of Casp. 8, Casp. 3, and PARP were analyzed in each group after treatment. All data were represented as mean ± SD (Two-way ANOVA, Tukey’s test, ns. p>0.05).

**Supplementary Figure 4:** **Procaspase-8 L491-F493 substitutions abolished apoptosis in 293T cells**.

Apoptosis (**A**) analysis stained with Annexin V-FITC and Δψ (**B**) analysis stained with JC-1 by flow cytometry in 293T cells expressing the Flag-tagged indicated proteins. (**C**) 293T cells expressing the Flag-tagged indicated proteins were treated with 100 nM etoposide or without for 8 hours, following flow cytometric analysis of Annexin V-FITC staining. Representative results were from 3 independent experiments, respectively.

**Tables**

**Supplementary Table 1: The clinic-pathological characteristics of patients without P10 mutations, including the WT patients and the non-P10 mutation patients (****without the identified P10 mutations but with other mutations in the P10 region)**

| **Variables** | | **non-P10 mutation** | **WT** | ***p* value** |
| --- | --- | --- | --- | --- |
| **Age** | < 60 y | 24 | 21 | 0.5102 |
|  | ≥ 60 y | 7 | 9 |  |
| **Gender** | Male | 17 | 20 | 0.3445 |
|  | Female | 14 | 10 |  |
| **WBC** | >10x10^^9^/L | 16 | 13 | 0.5174 |
|  | ≤10x10^^9^/L | 15 | 17 |  |
| **FAB classification** | M1 | 5 | 2 | 0.1617 |
|  | M2 | 6 | 11 |  |
|  | M3 | 9 | 5 |  |
|  | M4 | 6 | 10 |  |
|  | M5 | 1 | 2 |  |
|  | M6 | 3 | 0 |  |
|  | M7 | 1 | 0 |  |
| **Response to**  **Chemotherapy** | Remission | 8 | 12 | 0.2831 |
|  | Non-remission | 23 | 18 |  |

χ^2^ test, * p＜0.05

**Supplementary Table 2: The association between clinic-pathological features and specific mutations (FLT3-ITD ; CEPBA ; NMP)**

| **Variables** | | **FLT3-ITD mutations** | **Without FLT3-ITD mutations** | ***p* value** | **CEPBA mutations** | **Without CEPBA mutations** | ***p* value** | **NMP mutations** | **Without NMP mutations** | ***p* value** |
| --- | --- | --- | --- | --- | --- | --- | --- | --- | --- | --- |
| **Age** | < 60 y | 23 | 94 | 0.2908 | 21 | 96 | 0.7336 | 8 | 110 | 0.5257 |
|  | ≥ 60 y | 3 | 26 |  | 6 | 23 |  | 1 | 27 |  |
| **Gender** | Male | 12 | 72 | 0.1641 | 12 | 71 | 0.1494 | 8 | 78 | 0.0819 |
|  | Female | 14 | 46 |  | 15 | 48 |  | 1 | 59 |  |
| **WBC** | >10x10^^9^/L | 14 | 47 | 0.1688 | 16 | 67 | 0.7794 | 3 | 80 | 0.1414 |
|  | ≤10x10^^9^/L | 12 | 73 |  | 11 | 52 |  | 6 | 57 |  |
| **Response to**  **Chemotherapy** | Remission | 2 | 32 | 0.0415* | 7 | 26 | 0.6475 | 1 | 31 | 0.6840 |
|  | Non-remission | 24 | 88 |  | 20 | 93 |  | 8 | 106 |  |

χ^2^ test, * p＜0.05

**Supplementary Table 3: The association between the FLT3-ITD mutations and the P10 mutations**

|  | Without P10 mutations | P10 mutations | χ^2^ | *p* value |
| --- | --- | --- | --- | --- |
| FLT3-ITD mutations | 15 | 11 | 3.292 | 0.0696 |
| Without FLT3-ITD mutations | 46 | 74 |  |  |

χ^2^ test, * p＜0.05
