# Supplementary figures and images for: Mutations in the P10 region of procaspase-8 lead to chemotherapy resistance in acute myeloid leukemia by impairing procaspase-8 dimerization
Source: Cell Death Dis. 2018 May 3;9(5):516. doi: 10.1038/s41419-018-0511-3 (PMC5938697; doi:10.1038/s41419-018-0511-3)

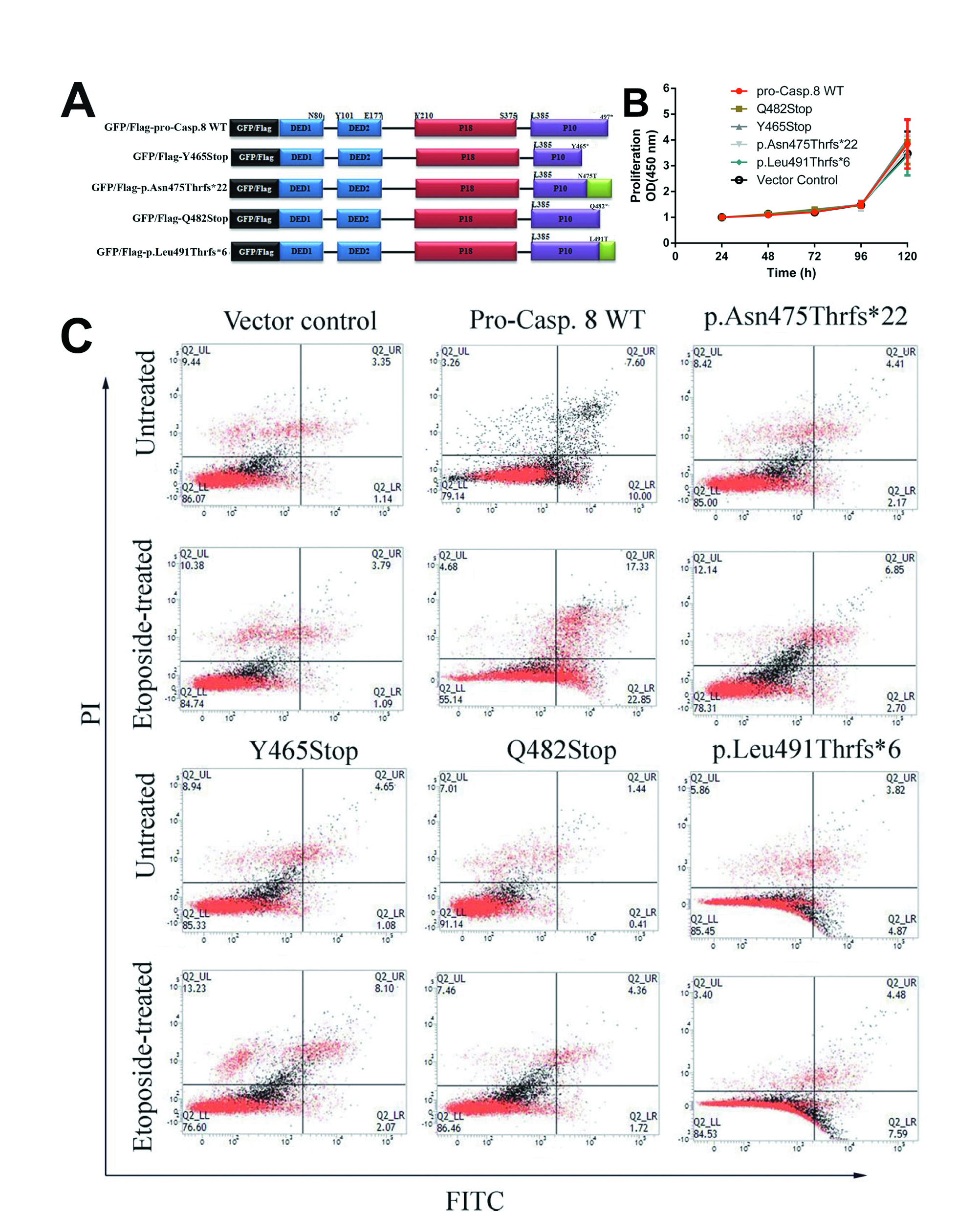

Supplement: Supplementary file 2 — supplementary Figure 1 [file 41419_2018_511_MOESM2_ESM.jpg]

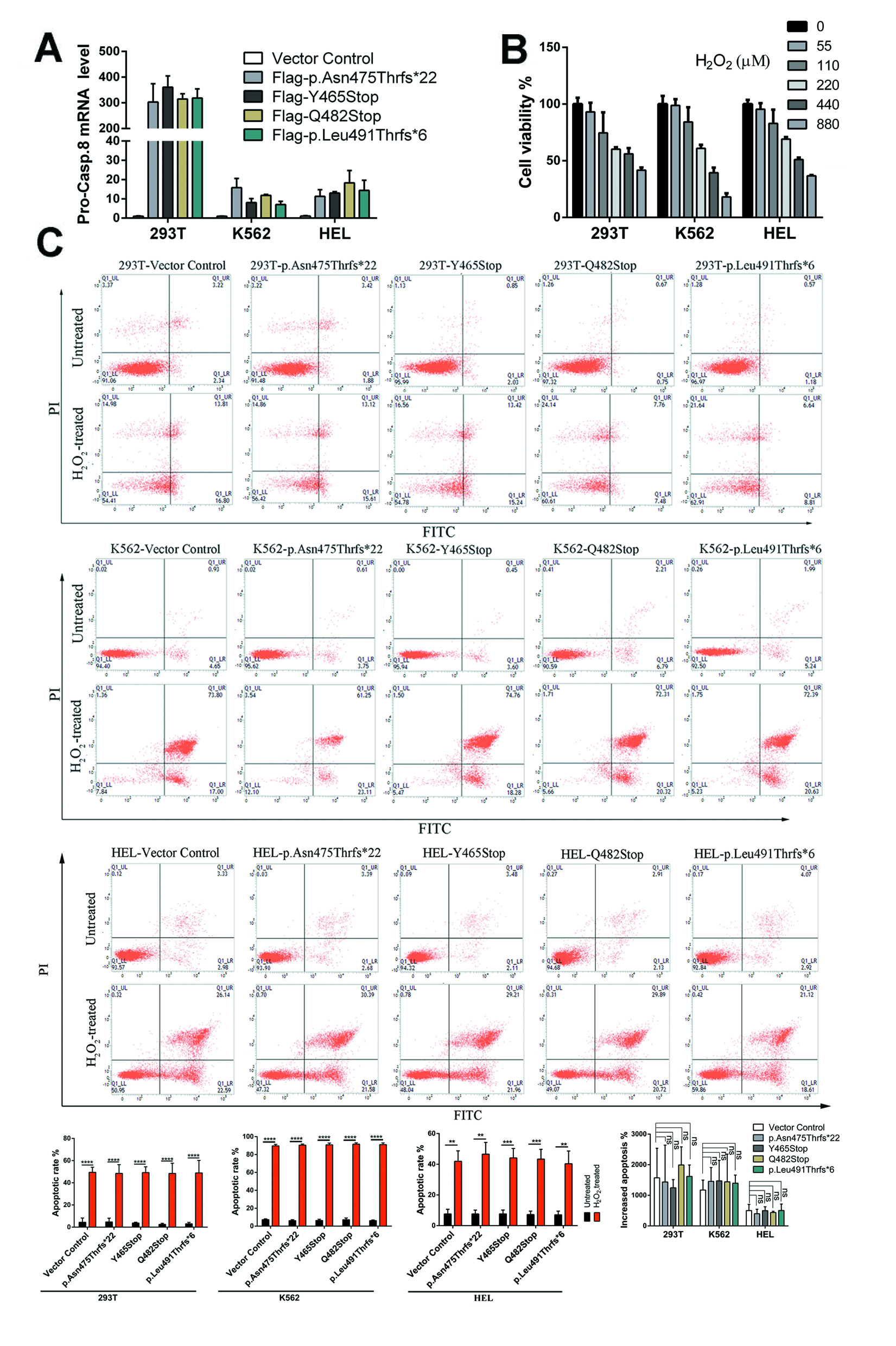

Supplement: Supplementary file 3 — supplementary Figure 2 [file 41419_2018_511_MOESM3_ESM.jpg]

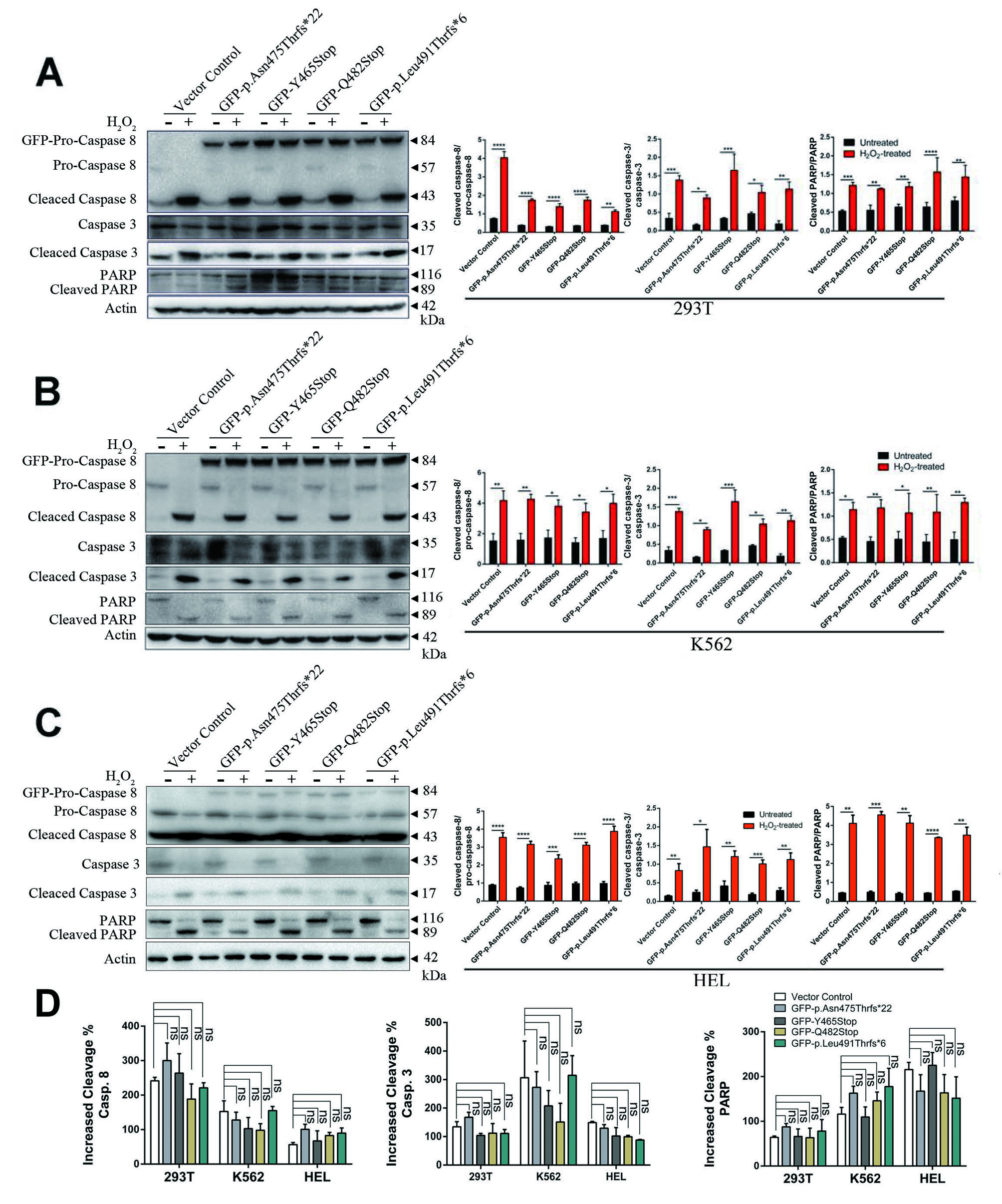

Supplement: Supplementary file 4 — supplementary Figure 3 [file 41419_2018_511_MOESM4_ESM.jpg]

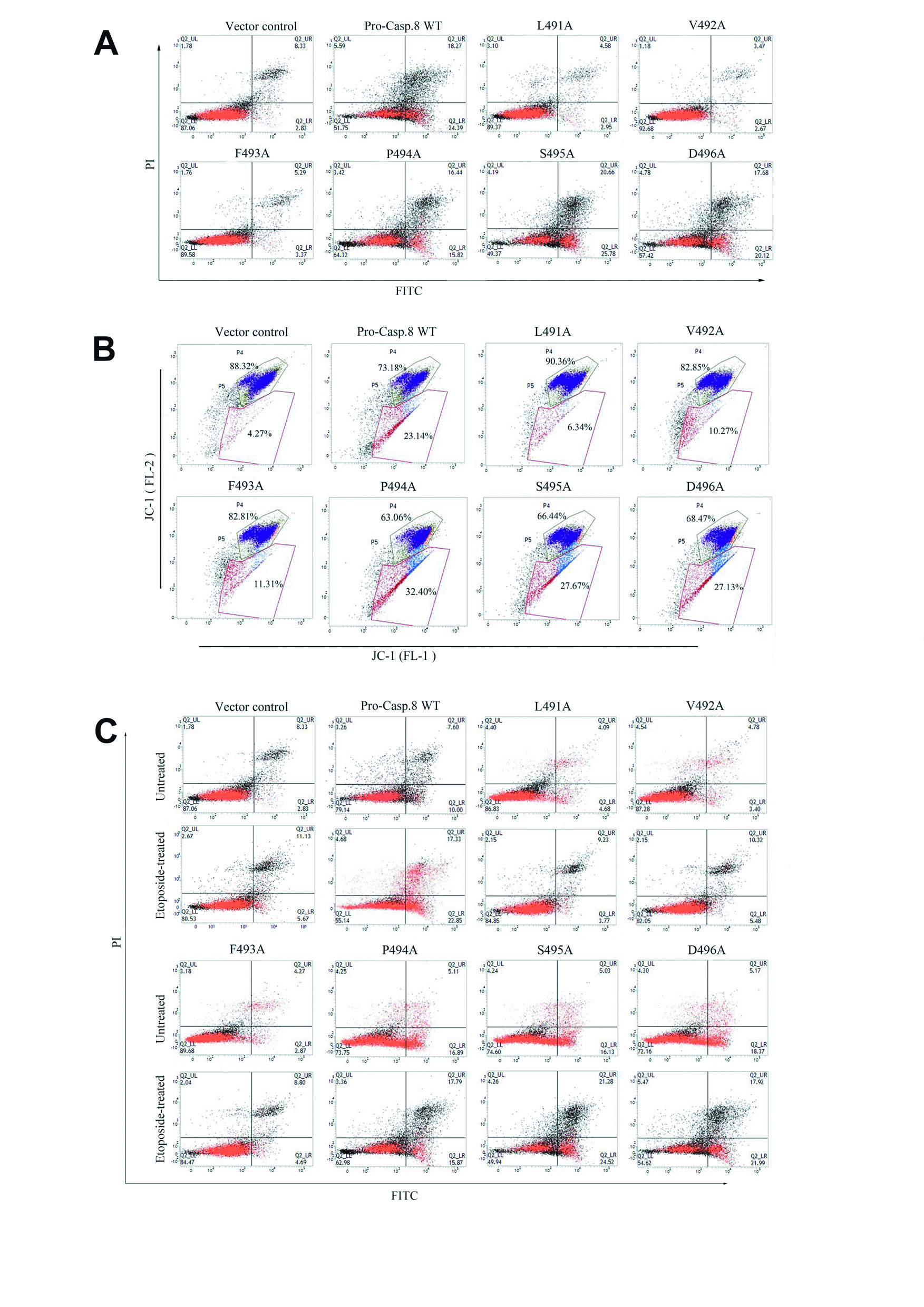

Supplement: Supplementary file 5 — supplementary Figure 4 [file 41419_2018_511_MOESM5_ESM.jpg]
